# Influence of Welding Control Mode on the Joint Performance of Ultrasonically Welded Carbon Fiber-Reinforced Polycarbonate

**DOI:** 10.3390/ma19061138

**Published:** 2026-03-14

**Authors:** Zhaolong Zhang, Yuanduo Yang, Lunan Wei, Sansan Ao, Yang Li

**Affiliations:** 1 School of Materials Science and Engineering, Tianjin University, Tianjin 300354, China; zhangzhaolong@tju.edu.cn (Z.Z.); yyd1111@tju.edu.cn (Y.Y.); ao33@tju.edu.cn (S.A.); 2 College of Civil Engineering, Anhui Jianzhu University, Hefei 230601, China; weilunan@ahjzu.edu.cn

**Keywords:** ultrasonic welding, CFRTP, CF/PC, welding control mode, welding quality

## Abstract

Carbon fiber-reinforced thermoplastic (CFRTP) composites are now widely used in many fields. Ultrasonic welding (UW) is a key technology for joining these materials. The control mode of UW has a great effect on the quality of the welded joints. However, there is still not enough research comparing the different welding control modes. This paper investigates the effects of the time control, energy control, and displacement control modes on the ultrasonic welding quality of carbon fiber-reinforced polycarbonate (CF/PC). A flat PC film is used as the energy director (ED). The evaluation focuses on the lap-shear strength (LSS), macro- and micro-morphology, fracture surface characteristics and power–displacement curves of the welding process. Furthermore, significant differences are observed in the temperature field evolution and joint failure modes across the different control modes and process parameters. Results indicate that the displacement control mode achieves the highest joint quality and process stability, yielding a maximum LSS of 30.6 MPa. A correlation analysis reveals that the displacement–energy relationship exhibits the strongest coupling, and the Pearson correlation coefficient r is 0.896.

## 1. Introduction

In recent years, driven by the rapid development of high-end manufacturing industries and increasingly stringent environmental regulations, high-performance carbon fiber-reinforced thermoplastics (CFRTPs) have been increasingly applied in the field of aerospace, automotive, electronics and so on [1,2,3,4,5,6]. Carbon fiber-reinforced polycarbonate (CF/PC), as a thermoplastic material featuring low price, high impact resistance and good heat resistance, has become one of the key candidates [7,8,9]. However, achieving an efficient and reliable joining of CF/PC components remains a critical challenge for manufacturing industries.

Ultrasonic welding is widely used in CFRTP assembly due to its high efficiency, clean process, and suitability for automation [10,11,12,13]. It can effectively avoid the stress concentrations induced by mechanical fastening while eliminating the long curing times, potential contamination, and process complexity associated with adhesive bonding [11,12,13,14]. However, that welding quality is highly sensitive to welding parameters and control modes such as welding time, energy input and welding displacement.

Schaefer et al. [15] conducted ultrasonic welding on polyamide (PA) and PC without using an energy director (ED) under the time control mode. The results showed that the moisture content was more important than energy input for the weld quality. Ren et al. [16] employed numerical simulations and experiments to characterize interfacial heating and bonding in CF/PC under the time control mode. Their work highlighted the influence of lap configuration on weld formation, achieving a lap-shear strength of 18 MPa without ED. Chinnadurai et al. [17] systematically examined the relationships between process parameters and joint strength in PC/ABS blends under the time control mode. The results reveal that the welding pressure and welding time have a significant impact on the weld strength. Bonmatin et al. [18] conducted research on the ultrasonic welding of carbon fiber/polyether ether ketone (CF/PEEK) with a PEI interface layer under the time control mode. The results showed that welding force and welding time were identified as the dominant parameters governing the interfacial temperature evolution and joint strength, whereas the influence of vibration amplitude was comparatively minor. Birtha et al. [19], in their spot-welding study on unidirectional tapes made of PC and carbon fibers under the time control mode, further identified welding temperature as a key factor governing weld formation and performance, and they achieved a tensile strength of approximately 23 MPa using a high-temperature (>350 °C), short-time (<2 s) welding strategy. Villegas [20] further proposed an in situ monitoring approach based on the power–displacement signals of the welding of CF/PEI thermoplastic composites under the time control mode to identify the interfacial melting state and determine the optimal welding window, confirming a strong correlation between process signals and joint strength. Pirondi et al. [21] compared the ultrasonic welding of continuous glass-fiber-reinforced PC (GF/PC) laminates under the energy control mode with and without an ED made of PC wire, confirming that the incorporation of an ED significantly improved the lap-shear strength and welding consistency. Liu et al. [22] indicated that an optimal welding displacement existed for the best welding quality of carbon fiber/polyetherimide (CF/PEI) and carbon fiber/epoxy (CF/epoxy) composites, whose value depended on the thickness of the ED. In which case, the level of the defects within the weld was minimized, and the joints failed cohesively within the composite substrates. Köhler et al. [23] conducted ultrasonic welding on unidirectional carbon fiber-reinforced thermoplastic composites under the displacement control mode. The results indicated that optimizing the ED coverage area at the interface could effectively suppress the resin squeeze-out. Villegas et al. [24] systematically studied the ultrasonic welding behavior of CF/PEI thermoplastic composites and the melting of the ED and the subsequent interfacial cooling–solidification process based on power–displacement signals. It was demonstrated that the displacement-controlled strategy can reliably maintain the welding process within the optimum melting stage, thereby producing welded joints with the highest strength and the lowest strength scatter. Palardy et al. [25] conducted ultrasonic welding on carbon fiber/polyetherimide (CF/PEI) with different flat ED thicknesses under various control modes. The results showed that for the 0.06 mm thick ED, the optimum weld quality is reached approximately at the onset of the displacement of the sonotrode. Therefore, displacement control could not be used. Instead, this quality could only be achieved through time or energy control modes. Zhao et al. [26] compared the effect of the time control mode, energy control mode, and displacement control mode on the ultrasonic welding of CF/PEEK, demonstrating that displacement control yields the highest welding consistency and the lowest strength scatter. Bhudolia et al. [11] systematically summarized the effects of control modes, ED design, amplitude, and welding pressure on melting behavior and joint strength, highlighting the critical roles of ED and displacement-controlled welding in achieving stable, high-strength joints. Goto et al. [27] conducted ultrasonic welding on CF/PA6 and found that the energy control mode demonstrated the high weld quality.

Although most studies indicated that the displacement control mode can achieve higher joint strength, many other studies suggested that the time or energy control modes perform better. This discrepancy in optimal control modes across different studies may be attributed to variations in resin types and fiber orientations. Currently, there are no studies that systematically compare the impact of welding control modes on the welding quality of CF/PC. In addition, the correlation of the three control modes is also insufficiently understood. Furthermore, the correlation between the three welding control modes has not been revealed. Therefore, this paper investigates the effect of welding control modes on the ultrasonically welded quality of CF/PC and performs a correlation analysis on the three control modes. The results are expected to provide theoretical support and process guidance for the high-performance joining of CF/PC components.

## 2. Experimental

### 2.1. Materials

The materials used in this paper were continuous carbon-fiber-reinforced polycarbonate (CCF/PC) laminates supplied by Nanjing Advanced Thermoplastic Composite Co, Ltd. (Nanjing, China). The laminates featured a 0/90 cross-ply stacking sequence with T300 carbon fibers and a fiber mass fraction of 55%. Each CCF/PC sheet with dimensions of 300 mm × 300 mm × 2 mm was made from 11 prepregs. Consolidation was carried out in a compression molding press at 385 °C under a pressure of 1 MPa for 30 min, resulting in a nominal laminate thickness of approximately 2.0 mm. After consolidation, the CCF/PC sheets were cut into samples with dimensions of 100 mm × 25 mm × 2 mm for subsequent ultrasonic welding experiments.

### 2.2. Ultrasonic Welding Procedure

**Figure 1:**
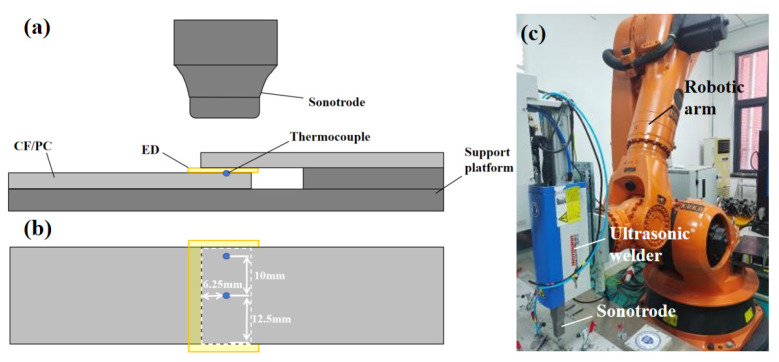
Ultrasonic welding experiment: (**a**) schematic of the ultrasonic welding setup; (**b**) schematic of thermocouple temperature measurement locations; (**c**) ultrasonic welding system.

Ultrasonic welding experiments were performed using a VE20 SL DIALOG 6200 ultrasonic welding system (Herrmann Ultraschalltechnik GmbH & Co. KG, Karlsbad, Germany) operating at a frequency of 20 kHz. A rectangular sonotrode with a tip area of 30 mm × 20 mm was employed. All samples were welded in a single-lap shear configuration with an overlap area of 25 mm × 12.5 mm. During welding, a 0.6 mm thick PC film was placed between the upper and lower adherends to act as the ED, as shown in Figure 1a.

Ultrasonic welding was conducted under the time control mode, energy control mode, and displacement control mode, respectively. The welding parameters are shown in Table 1. When using the time control mode, the welding time changed in the range of 0.6~2.1 s, and the welding force, amplitude and hold time remained constant. The welding energy and welding displacement were adaptively output from the machine, which cannot be manually set before welding. Similarity, the same applies to the energy control mode and displacement control mode. Multiple samples were welded for each group of welding parameters, and four of them were used for lap-shear tests, while three of them were employed for thermocouple-based temperature measurements. During each welding, the welding data (welding energy and welding displacement for the time control mode, welding time and welding displacement for the energy control mode, welding time and welding energy for the displacement control mode) were recorded, paired, and fitted to determine the correlations among welding time, energy input, and displacement. The optimal welding parameters under each control mode were determined by analyzing the joint strength.

### 2.3. Temperature Measurement

The temperature evolution at the center and edge regions of the welding interface during ultrasonic welding was monitored using K-type thermocouples (OMEGA CHAL-005, OMEGA Engineering, Inc., Norwalk, CT, USA). The thermocouples were precisely positioned at two representative locations in the welding area to characterize temperature changes: the center point is 12.5 mm and 6.25 mm away from the edge of the overlapping area, respectively, and the edge point is 2.5 mm away from the edge of the overlapping area, as shown in Figure 1b. The K-type thermocouples used in the experiment featured a wire diameter of 0.125 mm. The sample frequency is 100 Hz and the response time is 10 ms, which is much shorter than the welding time (0.6–3 s), rendering the thermal lag effect negligible. For temperatures above 0 °C, this K-type thermocouple has a measurement error of ±2.2 °C or ±0.75% of the measured temperature (provided by OMEGA official website).

### 2.4. Testing and Characterization

Lap-shear tests were conducted on a Wance universal testing machine (TSE105D, Shenzhen Wance Testing Machine Co., Ltd., Shenzhen, China) at a constant crosshead speed of 2 mm/min. For each welding condition, four specimens (*n* = 4) were tested for lap-shear strength to ensure reproducibility, and the average values along with standard deviations were reported. To further elucidate the joint formation process and welding quality, non-destructive testing (NDT) of the welded joints was performed using a Diondo D2 high-resolution micro-computed tomography (micro-CT) system (diondo GmbH, Hattingen, Germany). The fracture morphology was observed using a Thermo Fisher Scientific Quattro S field emission scanning electron microscope (FE-SEM) (Thermo Fisher Scientific, Waltham, MA, USA).

## 3. Results and Discussion

### 3.1. Effect of Welding Control Modes on the Weld Quality

**Figure 2:**
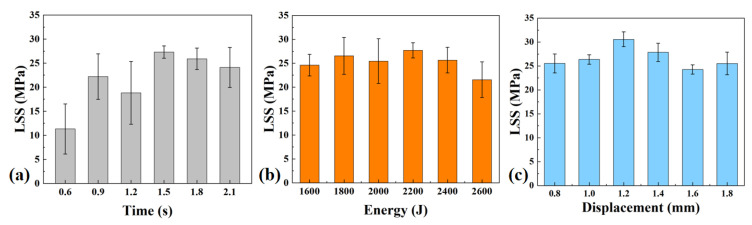
Lap-shear strength (LSS) of welded joints under different welding control modes: (**a**) time control mode; (**b**) energy control mode; (**c**) displacement control mode.

In this paper, the joint quality was evaluated in terms of lap-shear strength (LSS), as calculated by Equation (1).

(1) $$LSS=\frac{F}{S}$$

where *F* denotes the lap-shear force at joint fracture and *S* denotes the overlapping area (25 mm × 12.5 mm). The results are shown in Figure 2.

As shown in Figure 2a, the LSS exhibited pronounced strength fluctuations, as reflected by the relatively large error bars in the time control mode. The maximum LSS reached 27.3 MPa at the welding time of 1.5 s, whereas the minimum strength was only 11.3 MPa at the welding time of 0.6 s. These results indicate that under the time control mode, the energy input and interfacial consolidation state are difficult to maintain consistently, leading to significant variability in joint quality. Villegas et al. [24] also observed a similar phenomenon, noting that the time-control mode, due to the lack of feedback, failed to consistently terminate the process at the stage where maximum joint strength was achieved, resulting in an instability of joint quality.

As shown in Figure 2b, the energy control mode exhibited a relatively stable strength level across the investigated energy range. The overall scatter reflected by the error bars is noticeably lower than that observed under the time control mode. The joint strength reached a maximum of 27.7 MPa at a welding energy of 2200 J and a minimum of 21.6 MPa at 2600 J. These results suggest that using a fixed total energy input as the termination criterion provides more constant heat for interfacial melting, thereby leading to a more stable and consistent joint formation.

As shown in Figure 2c, the displacement control mode demonstrates the most prominent strength performance among the three control modes. The error bars indicate the smallest overall scatter, and the strength values are relatively concentrated across different displacement settings. The maximum strength reached 30.6 MPa at a displacement of 1.2 mm, which was the highest value among all three control modes, while the minimum strength was 24.3 MPa at 1.6 mm. These results suggest that using the actual interfacial collapse as the termination criterion more effectively matches the material response during welding, thereby resulting in a more stable and repeatable welding process.

To quantify the impact of welding control modes on the lap-shear strength (LSS) of CF/PC joints, a one-way ANOVA was conducted using OriginPro 2024 (10.1.0.178) software at a significance level of 0.05. With the control mode as the single factor and six parameters per level as observations, the analysis yielded an F-statistic of 9.48, exceeding the critical value of 3.13 (*p* < 0.001 < 0.05) based on Equations (2)–(7). This indicates statistically significant differences in LSS among the three control modes at a 95% confidence level. Such findings confirm the superiority of the displacement control mode in enhancing the mechanical performance of ultrasonic welded joints, which is consistent with the results of strength scatter analysis and microstructural characterization.

(2) $$SS_{T}=\sum x_{ij}^{2}-\frac{T^{2}}{N}$$

(3) $$SS_{A}=\sum \frac{T_{i}^{2}}{n_{i}}-\frac{T^{2}}{N}$$

(4) $$MS_{A}=\frac{SS_{A}}{df_{A}}$$

(5) $$MS_{E}=\frac{SS_{E}}{df_{E}}$$

(6) $$SS_{E}=SS_{T}-SS_{A}$$

(7) $$F=\frac{MS_{A}}{MS_{E}}$$

where *SS_T_* denotes the total sum of squares, *T* denotes the total sum of all data, *N* denotes the total number of data, *SS_A_* denotes the sum of squares among groups, *MS_A_* denotes the mean square among groups, *df_A_* denotes the degrees of freedom among groups, *MS_E_* denotes the mean square within groups, *SS_E_* denotes the sum of squares within groups, and *df_E_* denotes degrees of freedom within groups, respectively.

**Figure 3:**
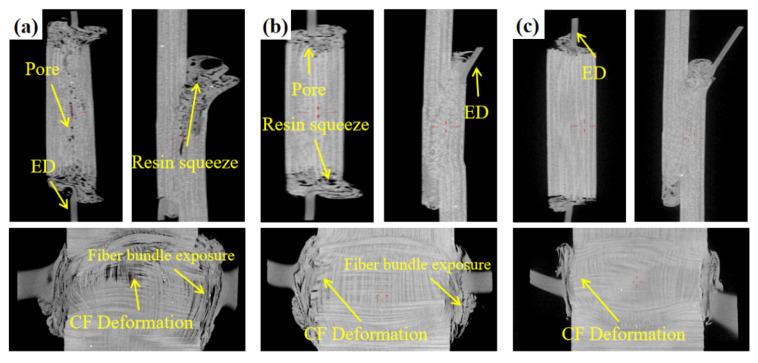
Non-destructive testing (NDT) images of welded joints under different welding control modes: (**a**) time control; (**b**) energy control; (**c**) displacement control.

To further compare the influence of welding control modes on weld quality, industrial computed tomography (CT) was employed to characterize internal defects in the welded joints of optimal parameters for each control mode (time = 1.5 s, energy = 2200 J, displacement = 1.2 mm), as shown in Figure 3. Pronounced differences in weld quality can be observed. Joints produced under the time control mode (Figure 3a) exhibited the poorest forming quality. Pronounced pores, extensive resin squeeze, fiber bundle exposure, and severe carbon fiber deformation were observed at the interface. These observations indicate that relying solely on the time control mode can cause severe mismatches between the energy input and interfacial consolidation state, thereby inducing significant welding defects.

Under the energy control mode (Figure 3b), although interfacial bonding was generally achieved, the inability to precisely match the energy input to the material requirements leads to excessive resin squeeze-out, fiber bundle exposure, a small amount of pores and carbon fiber deformation.

Under the displacement control mode (Figure 3c), the internal structure of the joint was relatively uniform with no apparent pore defects and resin squeeze-out. The interfacial bonding was dense. This indicates that controlled displacement enables stable interfacial pressure and molten resin flow between the adherends, effectively suppressing resin squeeze-out and thereby promoting the formation of high-quality joints.

### 3.2. Effect of Welding Control Modes on the Weld Formation Process

**Figure 4:**
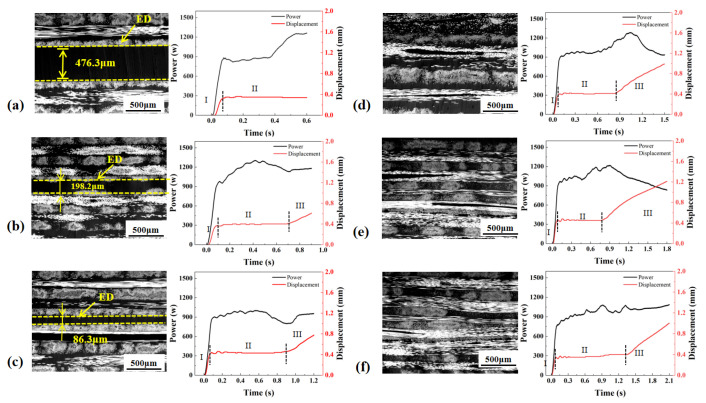
Power and displacement curves and morphologies of welded joints made with time control mode at the welding time of (**a**) 0.6 s; (**b**) 0.9 s; (**c**) 1.2 s; (**d**) 1.5 s; (**e**) 1.8 s; (**f**) 2.1 s.

**Figure 5:**
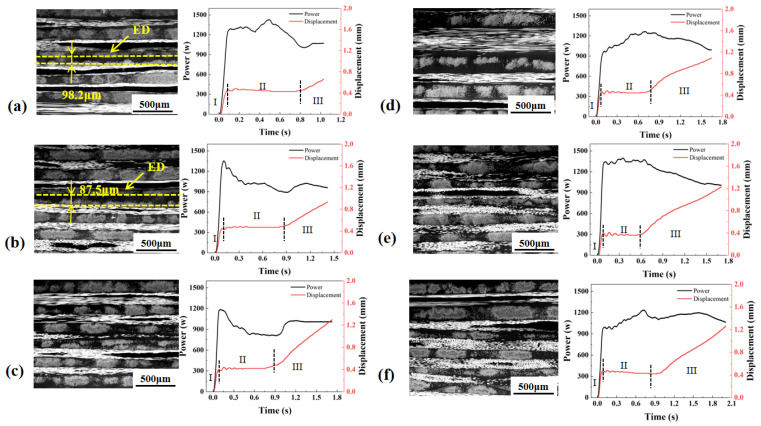
Power and displacement curves and morphologies of welded joints made with energy control mode at the welding energy of (**a**) 1600 J; (**b**) 1800 J; (**c**) 2000 J; (**d**) 2200 J; (**e**) 2400 J; (**f**) 2600 J.

**Figure 6:**
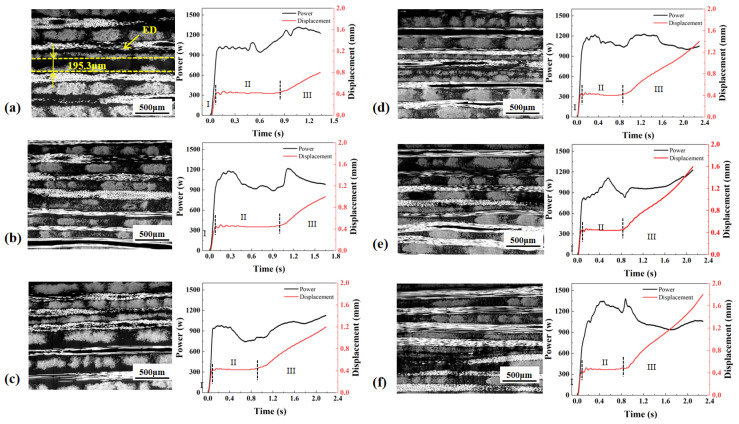
Power–displacement curves and morphologies of welded joints made with displacement control mode at the displacement of (**a**) 0.8 mm; (**b**) 1.0 mm; (**c**) 1.2 mm; (**d**) 1.4 mm; (**e**) 1.6 mm; (**f**) 1.8 mm.

Figure 4, Figure 5 and Figure 6 show the power and displacement curves and the morphologies of welded joints made with three control modes. The displacement curves under the three control modes exhibited consistent stage-dependent characteristics and can be uniformly divided into three stages according to the displacement evolution. In Stage I, the displacement increased rapidly, corresponding to the initial collapse and localized melting of the ED. In Stage II, the displacement entered a stable plateau, during which a continuous interfacial molten layer was progressively formed. In Stage III, the displacement increased slowly again, indicating further extrusion of the molten layer and sustained resin flow at the interface, leading to the gradual stabilization of the welded joint.

Under the time control mode (Figure 4), the ED retained a clear boundary at welding times of 0.6, 0.9 and 1.2 s, indicating incomplete melting under these process conditions. The total welding displacement was the smallest. The interface exhibited pronounced waviness and discontinuous regions, which was accompanied by large variations in molten-layer thickness. This indicates an insufficient collapse and compaction of the ED, leading to limited interfacial contact and restricted polymer-chain interdiffusion, and consequently resulting in the lowest joint strength. The power exhibited large fluctuations and was poorly synchronized with the displacement evolution, which readily caused local overheating or insufficient melting, resulting in unstable weld quality.

Under the energy control mode (Figure 5), the ED was not fully melted at 1600 J and 1800 J, where the ED boundary remained clearly observable at the interface. The interfacial continuity and molten-layer uniformity were markedly improved relative to the time control mode. However, due to fluctuations in the spatiotemporal distribution of energy input, the interfacial morphology still exhibited a certain degree of unevenness, yielding an intermediate strength level. Pronounced power variations still occurred with time, indicating non-uniform energy distribution at the interface.

Under the displacement control mode (Figure 6), incomplete melting of the ED can be observed only at the displacement of 0.8 mm, while at all other displacement levels, the ED was fully merged with the matrix resin, forming a continuous interfacial molten layer. The displacement control mode resulted in a total displacement comparable to that under energy control, while the displacement continued to increase slowly during Stage III. The interface was overall flatter and more continuous, the molten layer exhibited a more uniform thickness, and fiber bundles were more effectively embedded. These features indicate a more stable thermo-mechanical input and more sufficient healing, leading to the highest joint strength and the lowest strength scatter. The displacement control mode presented the most stable power evolution and was most closely coupled with the displacement evolution, accounting for the superior stability of the weld quality achieved under this mode.

Due to the inherent non-uniformity of displacement reduction across the overlapped region of the workpiece, the local thickness change in the ED observed in metallographic images is not entirely consistent with the global displacement variation recorded in the displacement curve. Table 2, Table 3 and Table 4 show the actual displacement (recorded by the ultrasonic welder), ED consumption (measured from the metallographic images) and net laminate penetration (the thickness difference between the initial laminate thickness and the post-welding thickness) in the three control modes. The net laminate penetration was measured at five representative positions (center and four corners of the overlap area), and the average value was used for quantification to ensure reliability. Equation (8) descripts the relationship between the actual displacement (S), ED consumption (C), and the net laminate penetration (N).

(8) S=C+N+E

Ideally, the system error (E) should be equal to zero. However, in this research, in all the cases, the E was about 0.4 mm. This is because the ultrasonic welder is mounted on a robotic arm, as shown in Figure 1c. Due to the inherent stiffness limitations of the robotic structure during operation, a portion of the displacement is absorbed by the arm’s elastic deformation, which generated a 0.4 mm system error. This phenomenon is quantitatively evidenced by the 0.4 mm plateau observed in the displacement–time curves.

Overall, among the three control strategies, the displacement control mode exhibited the highest capability to promote complete ED melting and stable interfacial formation, demonstrating the best interfacial formation stability. The energy control mode ranked second, whereas the time control mode showed the poorest stability, which is consistent with the LSS results presented in Figure 2.

**Figure 7:**
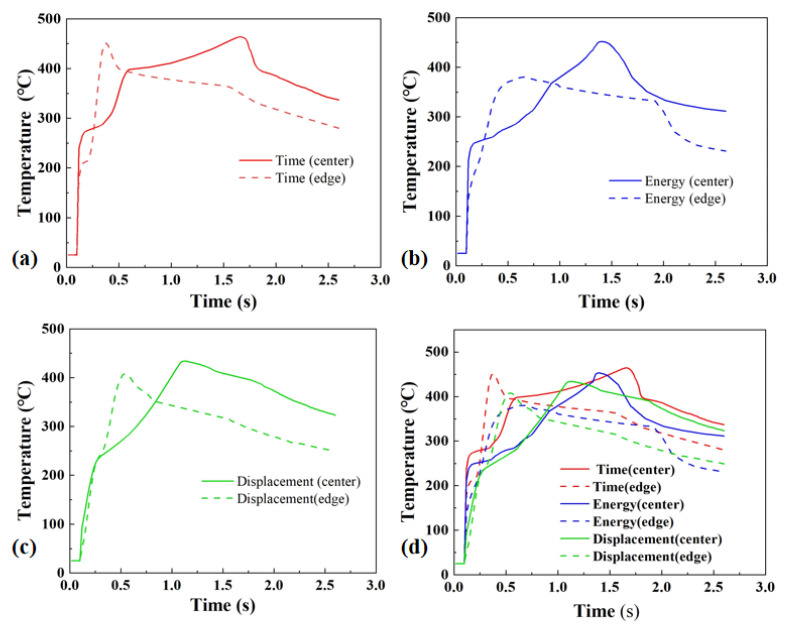
Representative temperature histories during welding under different control modes: (**a**) time control, time = 1.5 s; (**b**) energy control, energy = 2200 J; (**c**) displacement control, displacement = 1.2 mm; (**d**) comparison among time, energy, and displacement control modes.

A rational evolution of the temperature field is essential for achieving sufficient interfacial melting and the formation of high-strength welded joints. Figure 7a–c present temperature histories recorded during welding under the optimal parameters for the three control modes. Each temperature measurement curve is chosen as the most representative one from three replicate data sets. As indicated by the temperature curves, the temperature distribution within the joint during welding exhibited a distinct and consistent pattern. In the initial stage of welding, the temperature raised sharply, and the joint edge reached its peak temperature earlier than the center, indicating that heat was initially concentrated at the edge region and subsequently transferred toward the center. However, the peak temperature at the edge did not exceed that at the center. This behavior is primarily attributed to the edge effect, where frictional heat originates at the periphery and gradually propagates to the center as welding progresses. The resulting peak temperature difference between the center and the edge varies with the control mode, specifically 15 °C for the time control mode, 70 °C for the energy control mode, and 30 °C for the displacement control mode. This provides a fundamental basis for understanding the differences in joint formation under different control modes.

The peak interfacial temperature exceeded 400 °C, which is higher than the onset thermal degradation temperature of PC at 310–330 °C. However, no significant thermal degradation of the PC matrix was observed in the welded joints. McNeill et al. [28] reported that PC exhibits a major thermal decomposition peak near 460 °C. Temperatures around 400 °C only induce slow and slight decomposition under prolonged heating, and significant degradation requires continuous exposure to high temperatures for several minutes or longer. In the ultrasonic welding process, the duration during which the interfacial temperature exceeded 310 °C was only 1–5 s, which is too short to cause substantial decomposition.

Pronounced differences in temperature-field evolution are observed among the three control modes, as shown in Figure 7d. Under the displacement control mode, the temperature curves were relatively smooth, with a small temperature difference between the center and the edge, while the peak temperature remained at a moderate level without severe fluctuations, and the shortest time between the peak temperatures of the center and the edge was revealed. This indicates that the energy input under the displacement control mode is more effectively regulated. In contrast, under the energy control mode, larger temperature fluctuations were observed, which are accompanied by locally elevated temperatures and steep temperature gradients, and the time between the peak temperatures of the center and the edge was moderately extended, which may promote over-welded phenomena such as excessive resin extrusion and pore collapse in localized regions, thereby reducing joint stability. Under time control, the edge temperature raised rapidly at an early stage, while the center subsequently maintained a relatively high temperature over an extended plateau, and the longest time between the peak temperatures at the center and the edge was revealed. This mismatch in solidification sequence may adversely affect joint strength.

### 3.3. Effect of Welding Control Modes on Joint Failure Behavior

**Figure 8:**
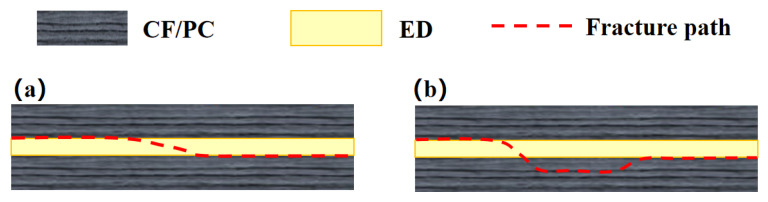
Schematic illustration of fracture morphologies: (**a**) interfacial fracture; (**b**) interlaminar fracture [29].

In this paper, the joint failure predominantly occurred in two typical modes: interfacial fracture propagating along the welding interfac, and interlaminar fracture deviating from the weld line and occurring within the parent laminate plies, as shown in Figure 8. Differences among the welding control modes, arising from their distinct approaches to regulating energy input and interfacial collapse, significantly affect the dominance and evolution of these two failure modes within the joints.

**Figure 9:**
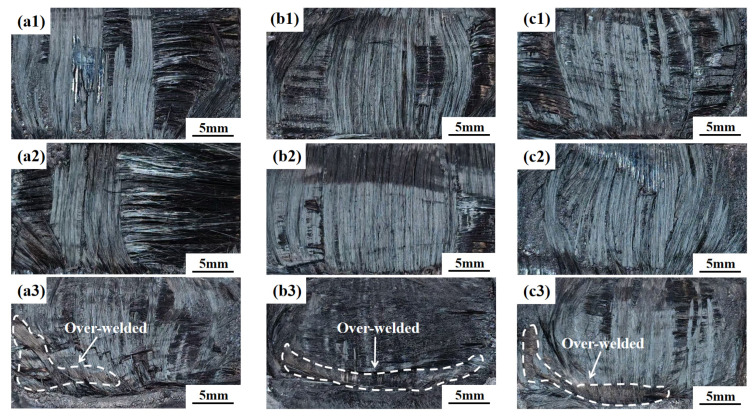
Macroscopic morphologies of welded joints: (**a1**) time = 1.2 s; (**a2**) time = 1.5 s; (**a3**) time = 2.1 s; (**b1**) energy = 1800 J; (**b2**) energy = 2200 J; (**b3**) energy = 2600 J; (**c1**) displacement = 0.8 mm; (**c2**) displacement = 1.2 mm; (**c3**) displacement = 1.8 mm.

As shown in Figure 9(a1–a3), under the time control mode, when the welding time was short, the degree of interfacial melting was limited, and joint failure predominantly occurred in the form of interfacial fracture. With increasing welding time, the interfacial resin gradually underwent sufficient melting and compaction, leading to a significant enhancement in interfacial bonding strength. Accordingly, the dominant failure mode transited to interlaminar fracture, which is accompanied by a pronounced increase in joint strength. When the welding time was further prolonged, the failure mode remained dominated by interlaminar fracture; however, excessive heat input induced over-melting and resin extrusion at the interface, reducing the effective load-bearing capacity of the weld region and consequently resulting in a decline in joint strength.

In contrast, under over-melting energy control mode (Figure 9(b1–b3)) and displacement control mode (Figure 9(c1–c3)), the failure behavior of each joint to parameter variations differs markedly from that observed under time control. Because the energy control mode directly regulates the total energy input and displacement control directly governs the interfacial collapse, sufficient interfacial melting and densification can be achieved even at relatively low parameter settings. As a result, joints produced under low energy or small displacement conditions already exhibited interlaminar fracture as the dominant failure mode. With further increase in welding energy or displacement, the failure mode remained primarily interlaminar fracture; however, the over-welded area became progressively more pronounced, leading to a reduction in the effective load-bearing cross-section of the weld and a corresponding decrease in joint strength.

**Figure 10:**
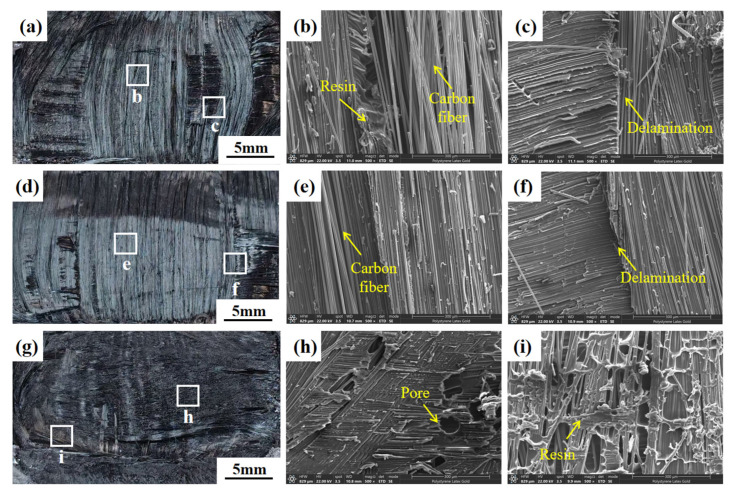
Micro-fracture morphologies of welded joints under displacement control mode: (**a**–**c**) displacement = 1.0 mm; (**d**–**f**) displacement = 1.2 mm; (**g**–**i**) displacement = 1.8 mm.

Specifically, the over-welded area was defined based on the following three criteria:

(1) Core quantitative index: net laminate penetration > 0.6 mm.
(2) Macroscopic criterion: distinct coked area was observed in the macroscopic morphology of the welded joints (Figure 9(a3,b3,c3)). The area was attributed to the thermal degradation and carbonization of polycarbonate resin caused by excessive heat input.
(3) Microscopic criterion: SEM observations revealed densely distributed pore defects in the weld zone, which are accompanied by resin flow accumulation and interfacial debonding between fibers and the resin matrix (Figure 10h,i).

Detailed fracture surface characterizations under the displacement control mode are revealed by SEM observation, as shown in Figure 10. It can be seen that when the displacement was small (1.0 mm), the fracture surfaces of the joints exhibited rough morphologies dominated by interlaminar fracture features. Resin residues and fiber pull-out were clearly observed at the interface, indicating insufficient interfacial melting and consolidation. Under optimal welding parameters (1.2 mm), the fracture surfaces became comparatively flat and smooth. A more uniform molten layer was formed between the resin matrix and the carbon–fiber surfaces, and the fracture surface predominantly exhibited a homogeneous interfacial fracture morphology, reflecting improved interfacial bonding quality. Under excessive displacement (1.8 mm), in addition to smooth interfacial fracture regions, localized over heating welding were observed, which is characterized by excessive resin flow and accumulation. This indicates that excessive melting, pore and resin extrusion occur at the interface, which may adversely affect the load-bearing capability of the welded joint. This also explains why 1.8 mm displacement was selected as the upper limit for the experiments.

The observed severe fiber deformation significantly reduces the reinforcement efficiency along the loading direction, preventing the fibers from attaining their maximum load-bearing capacity. Meanwhile, the exposure of fiber bundles signifies a loss of interfacial bonding between the fibers and the resin matrix, which hinders effective load transfer within the joint and induces severe stress concentrations. Such fiber damage fundamentally alters the paths of crack initiation and propagation. In an ideal interlaminar failure, fracture occurs within the plies of the parent laminate, typically indicating that the interfacial strength has exceeded the matrix strength. However, when the surface fibers at the interface are damaged or exposed, these regions act as weak links under mechanical load. Particularly in over-welded joints, although the failure manifests as interlaminar fracture, the structural integrity of the fiber bundles has been compromised by high temperatures during the welding process. Consequently, cracks propagate unstably and rapidly along the damaged fiber layers, leading to failure loads far below the theoretical values. Thus, while the failure mode seemingly transitions to interlaminar fracture, the corresponding joint strength suffers a significant decline.

### 3.4. Correlation Analysis Among Welding Control Modes

To evaluate the correlation among the three welding control modes, the optimal parameters for each mode were first determined based on the LSS. The other two associated parameters were also recorded during these optimal welding processes and then extracted. For instance, under the time control mode, the energy output and displacement at the optimal welding time were collected for analysis. To eliminate dimensional differences and ensure comparability, all extracted data were normalized using Equation (9) before the correlation analysis. After normalization, the three data sets (time, energy, and displacement) are mapped into the following interval [0, 1].

(9) $$D=\frac{X-X_{\min}}{X_{\max}-X_{\min}}$$

where X denotes the original data, and X_max_ and X_min_ represent the maximum and minimum values of the corresponding variable under a given control mode, respectively.

**Figure 11:**
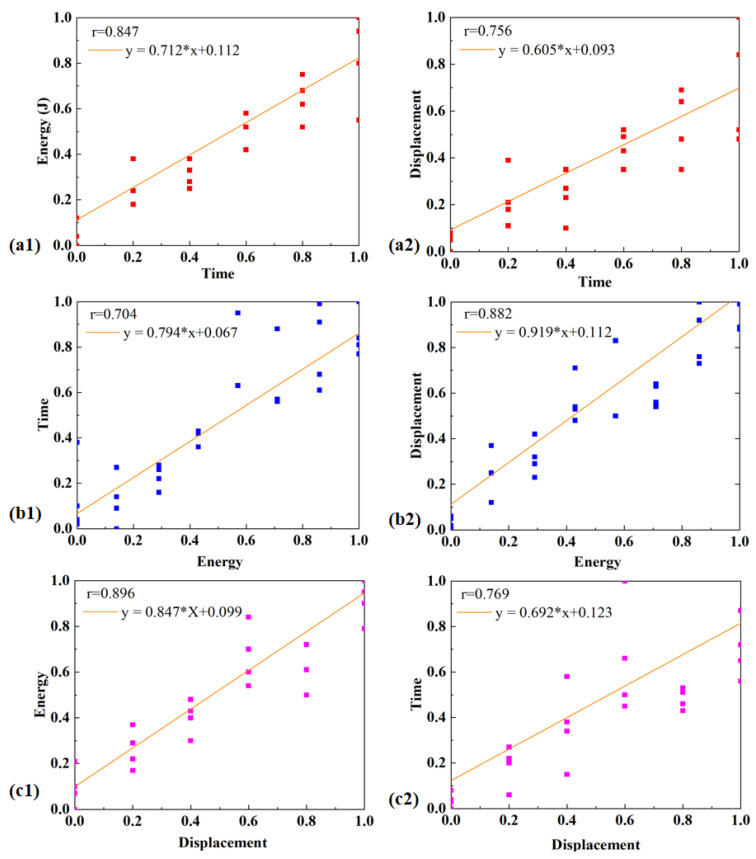
Scatter plots illustrating correlations among the three welding control modes: (**a1**) time–energy; (**a2**) time–displacement; (**b1**) energy–time; (**b2**) energy–displacement; (**c1**) displacement–energy; (**c2**) displacement–time.

The normalized data were then used to construct three sets of scatter plots (Figure 11), corresponding to the time–energy, time–displacement, and displacement–energy relationships. At a macroscopic level, the distributions of these scatter points exhibit approximately linear trends; however, the slopes and degrees of scatter differ among the three data sets, indicating pronounced differences in the coupling strength between variables under different welding control modes.

Based on the normalized scatter data, a univariate linear regression model was established using Equations (10)–(12):

(10) y=ax+b

(11) $$a=\frac{n\sum x_{i}y_{i}-\sum x_{i}{\sum y}_{i}}{n\sum x_{i}^{2}-{\left(\sum x_{i}\right)}^{2}}$$

(12) $$b=\frac{\sum y_{i}\sum x_{i}^{2}-\sum x_{i}\sum x_{i}y_{i}}{n\sum x_{i}^{2}-\sum x_{i}^{2}}$$

where *x* and *y* denote any two types of normalized control variables (e.g., time and energy, or displacement and energy). This approach ensures that the fitted line represents the optimal linear approximation in the least-squares sense.

From a physical perspective, different control variables evolve synchronously during the welding process, such as concurrent changes in molten-layer thickness, plastic collapse, and energy input. Thus, the corresponding scatter points will exhibit a stronger linear distribution. In such cases, linear regression can more accurately capture the coupling relationship between the variables.

After obtaining the fitted regression lines, the Pearson correlation coefficients among time–energy, time–displacement, and displacement–energy were further calculated using Equations (13)–(15). The analysis quantifies coupling between process variables:

(13) $$r=\frac{\sum_{i=1}^{n}\left(x_{i}-\bar{x})(y_{i}-\bar{y}\right)}{\sqrt{{\sum_{i=1}^{n}\left(x_{i}-\bar{x}\right)}^{2}-{\sum_{i=1}^{n}\left(y_{i}-\bar{y}\right)}^{2}}}$$

(14) $$\bar{x}=\frac{1}{n}\sum_{i=1}^{n}x_{i}$$

(15) $$\bar{y}=\frac{1}{n}\sum_{i=1}^{n}y_{i}$$

where *x* and *y* denote any two types of normalized control variables (e.g., time and energy, or displacement and energy).

Time–energy relationship: Based on the previously discussed power–displacement curves (Figure 4, Figure 5 and Figure 6), under the time control mode, the power peak and plateau regions are relatively stable, resulting in an energy accumulation process that is closer to a linear relationship. Consequently, a relatively high time–energy correlation coefficient (r = 0.847) is obtained (Figure 11(a1)). Under the energy control mode, transient power peaks are induced at the initial stage due to interfacial contact. As the molten layer develops and the interfacial thermo-mechanical response evolves, the system output power varies accordingly. These stage-dependent power fluctuations cause the energy input rate to deviate from constancy, thereby weakening the linearity of energy accumulation with time. As a result, the time–energy correlation coefficient under energy control decreases to r = 0.704 (Figure 11(a3)). This difference is consistent with the temperature-field analysis discussed earlier.

Time–displacement relationship: The previous analysis of interfacial formation indicates that displacement growth is primarily driven by the progressive collapse of the ED and interfacial melting. The power–displacement curves under all three control modes exhibit similar stage-dependent characteristics: rapid displacement increase at the initial stage, followed by a slower and more continuous growth during the middle and later stages. Consequently, time and displacement generally exhibit a moderate-to-strong linear relationship in terms of overall trend. As shown in Figure 11, the correlation coefficient under the time control mode is r = 0.756 (Figure 11(a2)), while that under the displacement control mode is r = 0.769 (Figure 11(c2)). Although these values are close, the correlation under displacement control is slightly higher. This is because under the displacement control mode, welding termination is directly governed by the actual interfacial collapse, allowing the process to better reflect the real interfacial state. Meanwhile, the relatively stable power output reduces excessive fluctuations, thereby decreasing the scatter in displacement evolution. This trend is also consistent with the earlier temperature-field and CT characterization results, which showed more stable temperature evolution and fewer interfacial defects under displacement control, leading to more consistent displacement development.

Displacement–energy relationship: The previously discussed temperature-field evolution, interfacial morphology, and CT inspection results all indicate that displacement growth primarily originates from interfacial melting and material deformation, which are directly driven by energy input. Therefore, the displacement–energy relationship reflects whether thermal input and interfacial deformation remain consistent during the welding process. Experimental results show that displacement and energy exhibit strong positive correlations under all three control modes. The correlation coefficient is r = 0.882 (Figure 11(b2)) for the energy control mode and 0.896 for the displacement control mode (Figure 11(c1)). Comparatively, the displacement control mode demonstrates the most pronounced correlation. This observation is in good agreement with the empirical findings discussed earlier: under displacement control, the power–displacement curves are more stable, the temperature evolution is smoother, the interfacial defects are minimized, and the final joint strength exhibits lower scatter.

However, due to the limited dataset, these results should be interpreted as observational trends specific to the CF/PC system rather than exhaustive statistical laws. Future work involving broader parameter ranges and confidence interval analysis would further refine these exploratory findings.

It is important to note that the findings of this study, particularly the superior performance of the displacement control mode, are based on a specific ED configuration (0.6 mm PC film). While displacement control demonstrates enhanced stability for this flat ED design, the optimal control strategy may shift with variations in ED geometry or thickness. Palardy et al. [25] demonstrated that for an extremely thin ED (0.06 mm), the displacement control mode was inapplicable; instead, optimal weld quality could only be achieved through time or energy control modes. Furthermore, Wang et al. [29] found that the time control mode performed more effectively for joints with embossed ED.

## 4. Conclusions

In this paper, the influence of the time, energy, and displacement control modes on the ultrasonic welding of CF/PC single-lap joints was systematically evaluated. The main results are given as follows.

(1) The displacement control mode demonstrated the highest joint quality and process stability. It achieved a maximum LSS of 30.6 MPa at 1.2 mm with the lowest data scatter. CT characterization showed a dense interface with minimal pores and no apparent resin squeeze-out. This is followed by the energy control mode, while the time control mode exhibits the lowest overall effectiveness for this material system.
(2) The ultrasonic welding process under all three control modes can be divided into three stages based on displacement evolution. The resulting peak temperature difference between the center and the edge varied with the control mode.
(3) Correlation analysis showed that the displacement–energy relationship had the strongest coupling with a correlation coefficient of r = 0.896 under the displacement control mode.
(4) Under the time control mode, the dominant failure mode transited from interfacial fracture at low parameters to interlaminar fracture as the welding time increased. Under the energy and displacement control modes, interlaminar fracture was the dominant failure mode even at low parameter settings.

## Figures and Tables

**Table 1:** Ultrasonic welding parameters.

Parameters	Value
Welding time (s)	0.6, 0.9, 1.2, 1.5, 1.8, 2.1
Welding energy (J)	1200, 1400, 1600, 1800, 2000, 2200
Welding displacement (mm)	0.8, 1.0, 1.2, 1.4, 1.6, 1.8
Welding force (N)	600
Amplitude (μm)	36.2
Hold time (s)	5

**Table 2:** ED consumption and net laminate penetration in the time control mode.

Set Welding Time (s)	Actual Displacement (mm)	ED Consumption (mm)	Net Laminate Penetration (mm)	System Error (mm)
0.6	0.51	0.12	0	0.39
0.9	0.80	0.40	0	0.40
1.2	0.92	0.51	0	0.41
1.5	1.42	0.60	0.41	0.41
1.8	1.61	0.60	0.59	0.42
2.1	1.74	0.60	0.73	0.41

**Table 3:** ED consumption and net laminate penetration in the energy control mode.

Set Welding Energy (J)	Actual Displacement (mm)	ED Consumption (mm)	Net Laminate Penetration (mm)	System Error (mm)
1600	0.92	0.50	0	0.42
1800	0.92	0.51	0	0.41
2000	1.21	0.60	0.21	0.40
2200	1.51	0.60	0.49	0.42
2400	1.57	0.60	0.58	0.39
2600	1.69	0.60	0.69	0.40

**Table 4:** ED consumption and net laminate penetration in the displacement control mode.

Set Displacement (mm)	Actual Displacement (mm)	ED Consumption (mm)	Net Laminate Penetration (mm)	System Error (mm)
0.8	0.8	0.4	0	0.40
1.0	1.0	0.6	0	0.41
1.2	1.2	0.6	0.21	0.40
1.4	1.4	0.6	0.39	0.41
1.6	1.6	0.6	0.62	0.41
1.8	1.8	0.6	0.79	0.42

## Data Availability

The original contributions presented in this study are included in the article. Further inquiries can be directed to the corresponding author.

## References

[1] Ikpe A.E., Itiat N.E., Ekanem I.I. (2025). A Systematic Review of Engineering Plastics and Their Viability in Conventional Industrial and Manufacturing Processes. J. Mater. Manuf. Technol..

[2] Vieyra H., Molina-Romero J.M., Calderón-Nájera J.D., Santana-Díaz A. (2022). Engineering, Recyclable, and Biodegradable Plastics in the Automotive Industry: A Review. Polymers.

[3] Nunes J., Silva J. (2016). Sandwiched composites in aerospace engineering. Advanced Composite Materials for Aerospace Engineering.

[4] Mallick P. (2021). Thermoplastics and thermoplastic–matrix composites for lightweight automotive structures. Design and Manufacturing for Lightweight Vehicles.

[5] Molinar-Díaz J., Parsons A.J., Ahmed I., Warrior N.A., Harper L. (2025). Poly-Ether-Ether-Ketone (PEEK) Biomaterials and Composites: Challenges, Progress, and Opportunities. Polym. Rev..

[6] Picard M., Mohanty A.K., Misra M. (2020). Recent advances in additive manufacturing of engineering thermoplastics: Challenges and opportunities. RSC Adv..

[7] Ozkan C., Karsli N.G., Aytac A., Deniz V. (2014). Short carbon fiber reinforced polycarbonate composites: Effects of different sizing materials. Compos. Part B Eng..

[8] Almushaikeh A.M., Alotaibi B.M., Alenad A.M., Alqahtani N.B., Alharbi A.G. (2021). Comprehensive Review of the Properties and Modifications of Carbon Fiber-Reinforced Thermoplastic Composites. Polymers.

[9] Kore S., Murthy V.S., Hiremath N., Theodore M., Young S., Penumadu D., Vaidya U. (2021). Textile-Grade Carbon Fiber-Reinforced Polycarbonate Composites: Effect of Epoxy Sizing. Ind. Eng. Chem. Res..

[10] Villegas I.F., Moser L., Yousefpour A., Mitschang P., Bersee H.E. (2012). Process and performance evaluation of ultrasonic, induction and resistance welding of advanced thermoplastic composites. J. Thermoplast. Compos. Mater..

[11] Bhudolia S.K., Gohel G., Leong K.F., Islam A. (2020). Advances in Ultrasonic Welding of Thermoplastic Composites: A Review. Materials.

[12] Li H., Chen C., Li Y.X., Wu J.J. (2022). Ultrasonic welding of fiber-reinforced thermoplastic composites: A review. Int. J. Adv. Manuf. Technol..

[13] Brito C.B., Teuwen J., Dransfeld C.A., Villegas I.F. (2025). Ultrasonic welding of thermoplastic composites: A comparison between polyetheretherketone and low-melt polyaryletherketone as resin in the adherends and energy directors. Compos. Part B Eng..

[14] Tsiangou E., Freitas S.T., Benedictus R., Villegas I.F. (2021). On the sensitivity of the ultrasonic welding process of epoxy- to polyetheretherketone (PEEK)-based composites to the welding force and amplitude of vibrations. Compos. Part C Open Access.

[15] Schaefer F.H. (2011). Ultrasonic Welding of Polyamide and Polycarbonate Plates. Key Eng. Mater..

[16] Ren Y.Y., Han B., Yan Q.Y. (2025). High-performance ultrasonic welding of continuous carbon fiber reinforced polycarbonate composites: Synergizing simulation and experimentation. Compos. Struct..

[17] Chinnadurai T., Arungalai V.S., Mohan R.N., Prakash N. (2017). Studies on ulteasonic welding of polycarbonate and acrylonitrile butasiene styrene blends. J. Chem. Technol. Metall..

[18] Bonmatin M., Chabert F., Bernhart G., Cutard T., Djilali T. (2022). Ultrasonic welding of CF/PEEK composites: Influence of welding parameters on interfacial temperature profiles and mechanical properties. Compos. Part A Appl. Sci. Manuf..

[19] Birtha J., Marschik C., Kobler E., Straka K., Steinbichler G., Schlecht S., Zwicklhuber P. (2023). Optimizing the Process of Spot Welding of Polycarbonate-Matrix-Based Unidirectional (UD) Thermoplastic Composite Tapes. Polymers.

[20] Villegas I.F. (2015). In situ monitoring of ultrasonic welding of thermoplastic composites through power and displacement data. J. Thermoplast. Compos. Mater..

[21] Pirondi A., Gulino M., Moroni F., Bercella M. (2024). Study of the ultrasonic welding of a polycarbonate-glass fiber laminate and comparison with adhesive bonding. Proc. Inst. Mech. Eng. Part L J. Mater. Des. Appl..

[22] Liu J., Yue D., Wang X., Pan J., Yang D., Quan D. (2024). Effects of welding displacement and energy director thickness on the ultrasonic welding of epoxy-to-polyetherimide based hybrid composite joints. Compos. Sci. Technol..

[23] Köhler F., Villegas I.F., Dransfeld C., Hermann A. (2021). Static ultrasonic welding of carbon fibre unidirectional thermoplastic materials and the influence of heat generation and heat transfer. J. Compos. Mater..

[24] Villegas I.F. (2014). Strength development versus process data in ultrasonic welding of thermoplastic composites with flat energy directors and its application to the definition of optimum processing parameters. Compos. Part A Appl. Sci. Manuf..

[25] Palardy G., Villegas I.F. (2017). On the effect of flat energy directors thickness on heat generation during ultrasonic welding of thermoplastic composites. Compos. Interfaces.

[26] Zhao Q., Wu H., Chen X., Chen X., Xu S., He C., Zhao T. (2023). Morphological Characterization and Failure Analysis of the Ultrasonic Welded Single-Lap Joints. Polymers.

[27] Goto K., Imai K., Arai M., Ishikawa T. (2019). Shear and tensile joint strengths of carbon fiber-reinforced thermoplastics using ultrasonic welding. Compo Part A Applied Sci. Manuf..

[28] McNeill I.C., Rincon A. (1991). Degradation Studies of Some Polyesters and Polycarbonates—8. Bisphenol A Polycarbonate. Polym. Degrad. Stab..

[29] Wang T., Zhang Z., Ao S., Wang K., Li Y. (2024). Ultrasonic welding of continuous carbon fiber reinforced PEEK with embossed energy directors. J. Manuf. Process..

